# Macular thickness varies with age-related macular degeneration genetic risk variants in the UK Biobank cohort

**DOI:** 10.1038/s41598-021-02631-2

**Published:** 2021-12-01

**Authors:** Rebecca A. Kaye, Karina Patasova, Praveen J. Patel, Pirro Hysi, Andrew J. Lotery, The UK Biobank Eye and Vision Consortium, Praveen J. Patel, Pirro Hysi, Andrew J. Lotery

**Affiliations:** 1 Clinical and Experimental Sciences, Faculty of Medicine, University of Southampton, Southampton, UK; 2 Department of Twin Research and Genetic Epidemiology, King’s College London School of Medicine, London, UK; 3 UCL Institute of Ophthalmology, National Institute for Health Research Biomedical Research Centre at Moorfields Eye Hospital NHS Foundation Trust and UCL Institute of Ophthalmology, London, UK; 4 Moorfields Eye Hospital, London, UK

**Keywords:** Medical research, Genetics research, Eye diseases, Retinal diseases

## Abstract

To evaluate the influence AMD risk genomic variants have on macular thickness in the normal population. UK Biobank participants with no significant ocular history were included using the UK Biobank Resource (project 2112). Spectral-domain optical coherence tomography (SD-OCT) images were taken and segmented to define retinal layers. The influence of AMD risk single-nucleotide polymorphisms (SNP) on retinal layer thickness was analysed. AMD risk associated SNPs were strongly associated with outer-retinal layer thickness. The inner-segment outer segment (ISOS)-retinal pigment epithelium (RPE) thickness measurement, representing photoreceptor outer segments was most significantly associated with the cumulative polygenic risk score, composed of 33 AMD-associated variants, resulting in a decreased thickness (p = 1.37 × 10^–67^). Gene–gene interactions involving the *NPLOC4-TSPAN10* SNP rs6565597 were associated with significant changes in outer retinal thickness. Thickness of outer retinal layers is highly associated with the presence of risk AMD SNPs. Specifically, the ISOS-RPE measurement. Changes to ISOS-RPE thickness are seen in clinically normal individuals with AMD risk SNPs suggesting structural changes occur at the macula prior to the onset of disease symptoms or overt clinical signs.

## Introduction

Age-related macular degeneration (AMD) is the leading cause of vision loss in high-income countries^1^, affecting more than 180 million people globally^2^. It is estimated that by the age of 75, approximately 30% of all Americans are affected by the disease^3^. AMD is a complex, progressive, chorioretinal degenerative disease that affects the macula, the central region of the retina. Three major factors contribute to AMD: advanced age, environmental and genetic risk factors^4–7^. Genetic studies have provided valuable insights into the mechanisms underlying AMD. Successful genome-wide association studies (GWAS) in AMD have led to the discovery of several key single nucleotide polymorphisms (SNPs) in genes conferring an increased disease risk^6,8^. The most recent comprehensive GWAS for AMD identified a total of 34 genomic loci that account for 46% of the genetic variance^6^. Due to high population frequency and effect sizes, SNPs in the cluster of genes *CFH-CFHR1-5* on chromosome 1, near the age-related maculopathy susceptibility 2 (*ARMS2*) and high-temperature requirement factor A1 *(HTRA1)* genes on chromosome 10 contribute nearly 80% of AMD's genetic risk^6,9–11^. The presence of at least one *CFH* risk allele alone is estimated to account for a population attributable risk fraction for early and late AMD of 10% and 53%, respectively^12^.

Although many genetic loci appear to confer risk for AMD development, the molecular pathophysiology behind such associations has not been fully elucidated. Furthermore, it is unknown if individuals carrying common risk polymorphisms display retinal phenotypes prior to the development of AMD clinical signs. A recent study examined the association of AMD susceptibility altering variants at *CFH-CFHR5* and *ARMS2/HTRA1* with macular retinal thickness in both normal individuals and those with AMD^13^. Their results showed thicker retinas in the perifovea for normal individuals with a protective *CFHR1/3* deletion, while eyes of *ARMS2/HTRA1* risk allele carriers with early or intermediate AMD had thinner retinas compared to those with *CFH-CFHR5* risk alleles. Whilst the focus of many genetic studies in AMD have been on the effects of chromosome 1 and 10 polymorphisms, including those surrounding retinal thickness^13,14^, the additional genetic loci identified in the aforementioned GWAS have not been further investigated, especially in normal individuals^6^.

Optical coherence tomography (OCT) imaging has revolutionised our understanding of retinal diseases, including AMD. Spectral-domain OCT (SD-OCT) imaging produces cross-sectional images of retinal layers using optical reflectivity differences between different layers of retinal cells from the retinal nerve fibre layer through to the retinal pigment epithelium. Segmentation software algorithms allow measurement of retinal layer thicknesses using differences in optical reflectivity to detect boundaries between retinal layers in vivo^15^. The UK Biobank is one of the largest prospective cohorts worldwide^16^, with a wealth of medical, lifestyle and detailed genetic sequencing data, including extensive data on ophthalmic diseases. This cohort provides the opportunity to investigate the impact of high-risk AMD genetic loci on changes in outer retinal layer thickness in clinically healthy participants from the UK Biobank population. This may provide mechanistic insight into how these genetic loci contribute to the development of AMD and identify novel biomarkers for clinical use.

## Methods

UK Biobank is a large-scale multisite cohort study that includes 502,682 participants, all residents of the United Kingdom, who were recruited via the National Health Service. The study was approved by the North West Research Ethics Committee (06/MRE08/65). Informed written consent was obtained from the participants. It was conducted according to the tenets of the Declaration of Helsinki.

The UK Biobank data resource was set up to allow detailed investigation of genetic and environmental determinants of major diseases of later life^16^. A detailed description of the study methodology has been published elsewhere^17^. Extensive baseline questionnaires, physical measurements, and biological samples were collected from participants at 22 assessment centres between 2006 and 2010^17^. Participants completed a touchscreen self-administered questionnaire on lifestyle and environmental exposures. The electronic questionnaire contained several inquiries about tobacco smoking habits, including past and current smoking status (UK Biobank Data Field number: 20116). After the initial baseline assessment, 23% (N = 117,279) of UK Biobank members also participated in an ophthalmic examination, a more comprehensive description of which can be found elsewhere^18,19^. A subset of this group (N = 67,321) also underwent spectral-domain optical coherence tomography (SD-OCT) scans.

Genotypes were available for most participants and their acquisition, imputation and quality control is described elsewhere^20^.

SD-OCT imaging was performed using the Topcon 3D OCT 1000 Mk2 (Topcon Corp., Tokyo, Japan) after visual acuity, autorefraction and IOP measurements were collected. OCT images were obtained under mesopic conditions, without pupillary dilation, using the 3D macular volume scan (512 A-scans per B-scan; 128 horizontal B-scans in a 6 × 6-mm raster pattern)^21,22^.

**Figure 1:**
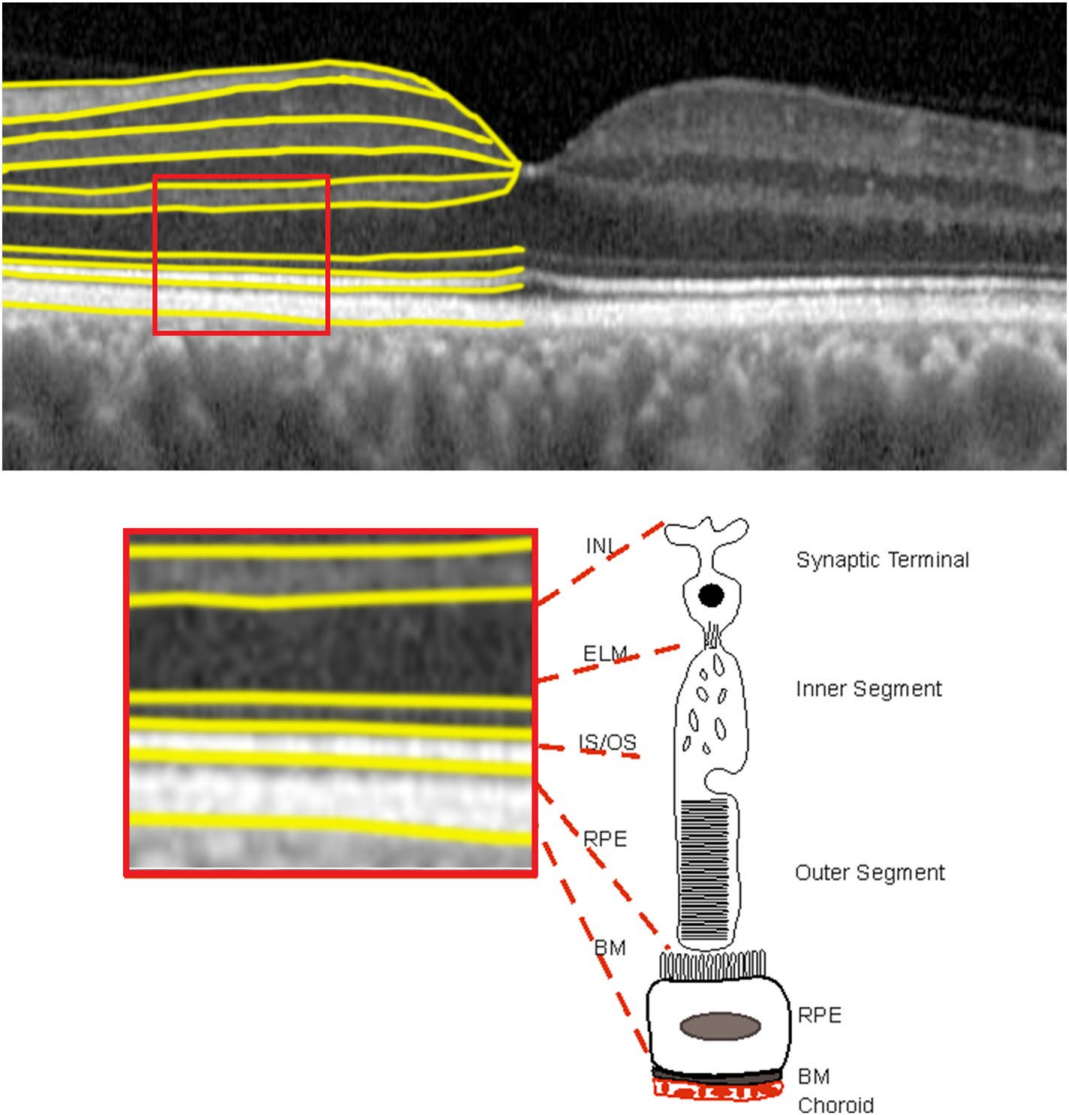
Optical coherence tomography with segmentation in half the image and a schematic demonstrating corresponding outer retinal layers in relation to photoreceptor segments and retinal pigment epithelium. Inner nuclear layer- External limiting membrane (INL-ELM) representative of the synaptic terminal. External limiting membrane—Inner and outer segments (ELM-ISOS) representative of the photoreceptor inner segment. Inner and outer segments—Retinal pigment epithelial thickness (ISOS-RPE), representative of the photoreceptor outer segment. Retinal pigment epithelium—ruch’s membrane (RPE-BM) representative of the RPE and BM complex.

Four SD-OCT measurements of outer retinal layer thickness were selected for our analyses of outer-retinal layer related boundaries as represented in Fig. 1: inner nuclear layer -retinal pigment epithelium (INL-RPE), retinal pigment epithelium-Bruch’s membrane (RPE-BM), and the specific sublayers of the photoreceptor: inner nuclear layer-external limiting membrane (INL-ELM); external limiting membrane-inner segment outer segment (ELM-ISOS); and inner segment outer segment-retinal pigment epithelium (ISOS-RPE)^23,24^. The accuracy of the segmentation is described here^25^. Additional details on how we used the algorithm to segment UKBB images are described here^22,23^. Briefly, the segmentation method includes an automated measure of signal strength, image centration and segmentation failure. In line with our previous work we defined poor image quality as an image with a signal strength of < 45 measured using Version 1.6.1.1 of the Topcon Advanced Boundary Segmentation (TABS) algorithm^25^. This algorithm is available upon request from Topcon Medical Limited. All segmentation measurements were calculated up to, but not including, the boundary layer. The TABS segmentation algorithm was used to segment the outer retinal layers^22,25^. The INL-ELM is a proxy measure of the synaptic terminal of the photoreceptor. The ELM-ISOS is representative of the photoreceptor inner segment. The ISOS-RPE measurement is representative of the photoreceptor outer segment. The RPE-BM measurement represents the RPE and BM complex. The anatomy of the outer retinal layers corresponds with the OCT boundaries observed in the retina (Fig. 1), hence the layers have been defined using the above specific definitions.

Two measurements were collated for each outer retinal layer, average and central thickness. Central thickness measurements represent the central 1 mm subfield area of the SD-OCT scan, corresponding to the fovea. Average measurements represent the mean thickness of outer retinal layers in the macula.

**Figure 2:**
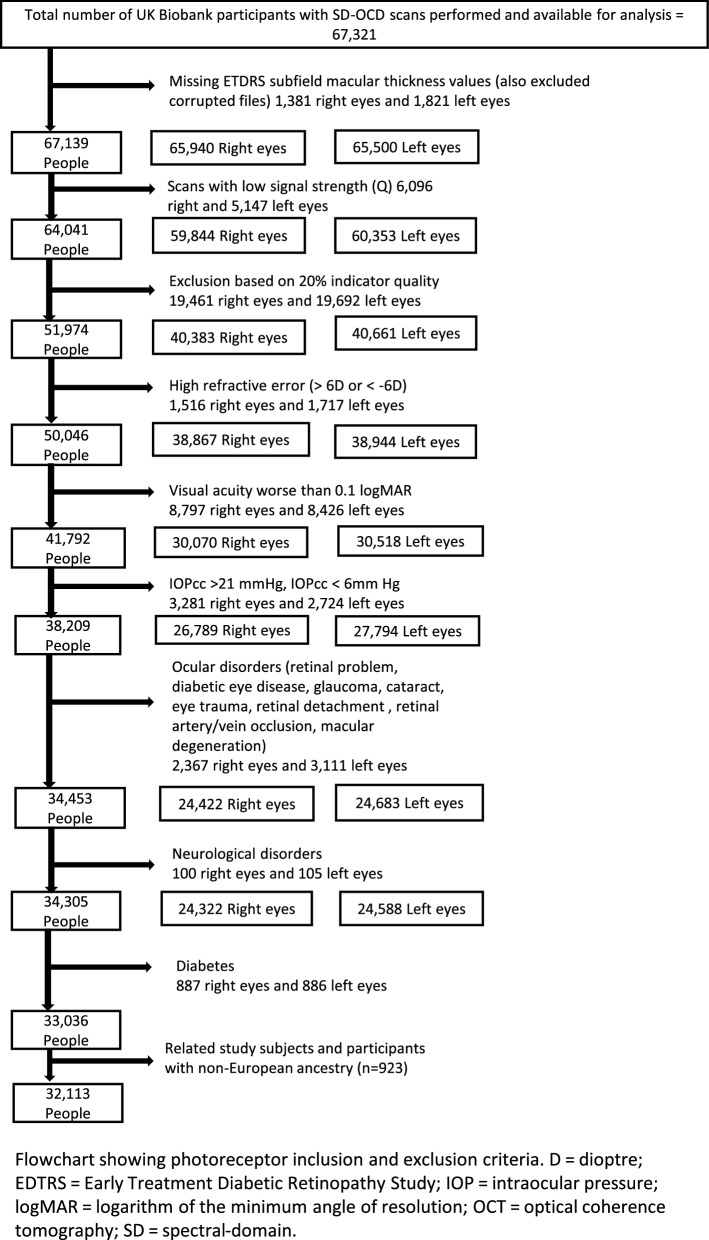
3D Bar Graph depicting the Inner nuclear layer-External limiting membrane (INL-ELM) (average) layer thickness and the additive effects of NPLOC4-TSPAN10 and CFH risk alleles. Alleles shown are those that confer additional AMD risk. The colour of the bars represents the increasing INL-ELM average layer thickness. Homozygosity for NPLOC4-TSPAN10 AMD-risk SNP (TT) rs6565597 and CFH protective SNP rs10922109 (AA) alleles revealed a significantly thinner INL-ELM. Absence of NPLOC4-TSPAN10 risk SNPs in the presence of homozygosity for the CFH protective SNP resulted in a significantly thickened INL-ELM. Homozygosity for NPLOC4-TSPAN10 AMD-risk SNP (TT) rs6565597 and CFH risk SNP rs10922109 (CC) alleles revealed a significantly thicker INL-ELM (p = 0.0004; adjusted p-value = 0.08).

Inclusion and exclusion criteria: The data set used was the same as described in Chua et al*.*^26^, therefore our exclusion criteria were equivalent. Briefly, all participants of European ancestry who underwent SD-OCT as part of the UK Biobank data collection were included in the initial analysis. To reduce potential confounding factors emerging from population genetic structure, the study sample was restricted to unrelated individuals of European descent. European ancestry was verified using genetic data from study participants. Exclusion criteria included participants who withdrew their consent, had poor SD-OCT signal strength, missing thickness values from any Early Treatment Diabetic Retinopathy Study (ETDRS) subfield, image quality score < 45, poor centration certainty, or poor segmentation certainty using TABS software^24,25^. We also excluded SD-OCT measurements by removing values outside 3 standard deviations. Participants with the following eye conditions were also excluded from the study: refractive error ± 6 dioptres (D); visual acuity worse than 0.1 logMAR; IOPcc of < 6 mmHg or > 21 mmHg; self-reported AMD or a recorded AMD diagnosis (ICD10 code), self reported glaucoma or ocular disorders. Patients with diabetes, neurodegenerative disease or diabetic neuropathy were also excluded. Figure 2 shows how many people were available after applying these exclusion criteria. For the purposes of our analyses we extracted the genotypic information for all loci associated with AMD described elsewhere^6^; high quality information was obtained for 33/34 such SNPs, because no high quality genotypes were available in the UK Biobank for the rarer rs142450006 SNP.

### Statistical analyses

Descriptive analyses were conducted using *epiDisplay* package in R (https://www.r-project.org/). Means and standard errors were calculated for normally distributed continuous variables. Categorical variables were characterized by computing frequencies and percentages.

To test the associations between selected AMD markers and outer retinal layer thickness measurements, we built linear models adjusted for age, sex, refraction and smoking habits of the study participants. In addition, we also computed polygenic risk scores (PRS) of AMD using alleles and effect and built linear models to assess PRS association with measurement of outer retinal layer thickness.

To further explore the possibility of an interaction between AMD risk-altering genotypes and their potential to non-linearly influence outer retinal layer thickness, we built linear regression models that, in addition to the above parameters, also included a genetic interaction (GI) term and individual AMD variants as independent predictors and each of the four SD-OCT measurements (ELM-ISOS, ISOS-RPE, INL-ELM and RPE-BM), as outcomes. Each of the average and central SD-OCT measurements was respectively tested for the unique pairwise combinations of SNPs that were previously found in association with any of the SD-OCT parameters beyond Bonferroni multiple testing correction. The latter was conservatively defined on the basis of 33 SNPs and 15 unique pairs of SNPs assessed in the GI analyses, multiplied by the number of SD-OCT measurements. We did not consider central and average measurements to be independent.

To further explore the relationship between AMD and outer retinal layer thickness measurements, we built Mendelian Randomization models (MR), testing potential causal associations between AMD and four different measurements of outer retinal layer thickness.

While regression tests generally are usually very powerful statistical tests aimed at identifying associations between two variables, they are not useful at determining the nature of that relationship. The association between two variable can arise as a consequence of a causal effect of any of the two variables on the other, or values of both variables may be determined by other known or unknown factors, often not even included in the model. Mendelian randomization are tests that specifically check direction of causation in the relationship between two associated variables. Mendelian randomization is a relatively new group of cross-sectional causal inference statistical methods^27,28^. Mendelian randomization tests the hypothesis that one particular trait (called an “exposure” in this context), whose variance is at least partially explained by the effect of known genetic loci (“instruments”), causally influences certain phenotypic expressions of another trait or disease (called “outcome”). These methods mimic randomised trials, effectively comparing individuals who are randomized based on the presence of high or low load of alleles predisposing towards the exposure, which under Mendel’s law of segregation is random and not affected by the presence of the outcome phenotype.

In this study, we chose as instrumental variables genetic markers that independently (r^2^ < 0.1) located in each genomic region associated with AMD. These instrumental variables were selected from previously published AMD GWAS^6^. The effect of these genes is a good approximation of the subsequent likelihood of AMD in individuals who currently present no objective signs of the disease. To minimize bias, we removed variants that showed substantial associations with SD-OCT parameters. To establish causality, we performed two types of experiments: first, where AMD-associated makers were used as the exposure variable, and SD-OCT measurements were the outcome, and then the inverse experiment where variants associated with outer retinal layer thickness served as an exposure variable and AMD was the outcome. The two-sample analyses were conducted using *MendelianRandomization* package in R. Several MR tests, including “simple median”, “inverse‐variance weighted”, and “MR-Egger”, were applied to assess causality. These tests are mutually complementary, and their results are usually interpreted together^29^.

## Results

The final study sample included 32,113 UK Biobank participants of European ancestry; 48% were men, with a mean age of 57 (± 8) years, and 54% of participants self-reported as ‘never smoked’. More detailed information regarding the demographic characteristics of the study subjects can be found in Supplementary Table S1. The distribution of outer retinal thickness measurements for INL-ELM, ELM-ISOS and ISOS-RPE, resembled a normal distribution; however, RPE-BM thickness followed a leptokurtic distribution (Supplementary Fig. S1).

We first tested the association between AMD susceptibility-increasing alleles and outer-retinal layer thickness measurements as described in the Methods. We identified several nominally significant associations with average SD-OCT measurements (Table 1, Supplementary Table S2), of which 10 remained significant after multiple testing corrections (p < 0.0004 (0.05/(4 × 33). The strongest association (p = 3.34 × 10^–54^) was observed between rs3138141, a variant located near the boundary between an intron and the third exon of the retinol dehydrogenase 5 (*RDH5*) gene and the average INL-ELM layer thickness (Table 1). Very significant associations were also found between rs10922109 within the *CFH* gene (p = 1.47 × 10^–49^) and rs6565597 within the *NPLOC4-TSPAN10* gene (p = 1.00 × 10^–38^) and RPE-BM average layer thickness (Table 1).

**Table 1:** Significant associations between average SD-OCT measurements and AMD variants.

Chr	Variant	Gene	EA	NEA	*ELM-ISOS*			*INL-ELM*			*ISOS-RPE*			*RPE-BM*		
					Beta	SE	p-value	Beta	SE	p-value	Beta	SE	p-value	Beta	SE	p-value
1	rs10922109	CFH	C	A	0.018	0.011	0.1	− 0.036	0.048	0.5	− 0.26	0.03	6.63 × 10^–20^	− 0.322	0.022	1.47 × 10^–49^
6	rs429608	PBX2	G	A	0.022	0.015	0.1	0.017	0.065	0.8	− 0.19	0.04	6.08 × 10^–07^	− 0.08	0.03	0.01
6	rs943080	VEGFA	T	C	− 0.003	0.011	0.8	− 0.048	0.047	0.3	0.005	0.03	0.9	− 0.084	0.021	8.83 × 10^–05^
7	rs7803454	PILRA	T	C	0.029	0.014	0	0.199	0.059	0.0008	0.245	0.04	5.15 × 10^–12^	− 0.107	0.027	7.67 × 10^–05^
9	rs10781182	RORB	T	G	0.001	0.012	1	0.215	0.051	2.11 × 10^–05^	− 0.05	0.03	0.1	0.024	0.023	0.3
10	rs3750846	ARMS2	C	T	0.014	0.013	0.3	0.074	0.057	0.2	− 0.26	0.03	5.32 × 10^–14^	− 0.068	0.026	0.01
12	rs3138141	BLOC1S1-RDH5	A	C	0.049	0.012	0.0001	0.824	0.053	3.34 × 10^–54^	0.389	0.03	2.44 × 10^–34^	0.091	0.024	0.0002
14	rs61985136	RAD51B	T	C	0.027	0.011	0.02	0.223	0.049	5.35 × 10^–06^	0.038	0.03	0.2	− 0.052	0.022	0.02
17	rs6565597	NPLOC4-TSPAN10	T	C	− 0.039	0.015	0.0008	− 0.374	0.05	8.76 × 10^–14^	0.377	0.03	4.25 × 10^–36^	− 0.3	0.023	1.00 × 10^–38^
19	rs2230199	C3	G	C	− 0.019	0.014	0.2	− 0.061	0.058	0.3	0.16	0.04	5.04 × 10^–06^	0.042	0.027	0.1

Columns “Variant”, “EA” and “NEA” list variants that were included in the model, and their risk alleles (EA, effect alleles for which the effect sizes are reported and NEA, non-effect alleles). Fields “Beta”, “SE” and “P” denote the change in SD-OCT measurements, standard errors and p-values of observed associations. The table includes significant linear regression results for 32,113 unrelated participants from UK Biobank participants. Models were adjusted for sex, age, spherical equivalent, smoking status.

We also identified associations with central SD-OCT measurements (Table 2, Supplementary Table S3). The statistically strongest association was found between ISOS-RPE layer thickness and the *RDH5* variant rs3138141 p = 7.21 × 10^–46^). Additionally, we also observed a strong association between the same marker (rs3138141; p = 1.22 × 10^–44^) and INL-ELM layer thickness. We found a significant association between RPE-BM layer thickness and the variant rs6565597 (p = 5.12 × 10^–45^) located in the intergenic region between *NPLOC4* and *TSPAN10* genes.

**Table 2:** Significant associations between central SD-OCT measurements and AMD variants.

Chr	Variant	Gene	EA	NEA	*ELM-ISOS*			*INL-ELM*			*ISOS-RPE*			*RPE-BM*		
					Beta	SE	p	Beta	SE	p	Beta	SE	P	Beta	SE	p
1	rs10922109	CFH	C	A	0.061	0.015	2.94 × 10^–05^	0.016	0.075	0.8	− 0.601	0.043	3.45 × 10^–44^	− 0.330	0.034	5.02 × 10^–22^
4	rs10033900	CFI	T	C	0.025	0.014	0.08	0.023	0.074	0.8	− 0.168	0.042	7.50 × 10^–05^	0.032	0.034	0.3
6	rs429608	PBX2	G	A	0.012	0.020	0.6	− 0.101	0.102	0.3	− 0.450	0.059	1.92 × 10^–14^	− 0.030	0.047	0.5
7	rs7803454	PILRA	T	C	− 0.017	0.018	0.4	0.187	0.094	0.05	0.127	0.054	0.02	− 0.190	0.043	8.38 × 10^–06^
10	rs3750846	ARMS2	C	T	0.047	0.018	0.01	0.102	0.090	0.3	− 0.682	0.052	2.36 × 10^–39^	0.011	0.041	0.8
12	rs3138141	BLOC1S1-RDH5	A	C	− 0.032	0.016	0.05	1.178	0.084	1.22 × 10^–44^	0.686	0.048	7.21 × 10^–46^	0.129	0.038	0.001
14	rs61985136	RAD51B	T	C	0.048	0.015	0.001	0.324	0.077	2.93 × 10^–05^	0.023	0.045	0.6	− 0.027	0.035	0.4
17	rs6565597	NPLOC4-TSPAN10	T	C	− 0.061	0.015	8.08 × 10^–05^	− 0.388	0.079	9.67 × 10^–07^	0.407	0.046	4.20 × 10^–19^	− 0.509	0.036	5.12 × 10^–45^
19	rs2230199	C3	G	C	− 0.029	0.018	0.1	− 0.068	0.092	0.5	0.275	0.053	2.15 × 10^–07^	0.003	0.042	0.9

Columns “Variant”, “EA” and “NEA” list variants that were included in the model, and their risk alleles (EA, effect alleles for which the effect sizes are reported and NEA, non-effect alleles). Fields “Beta”, “SE” and “P” denote the change in SD-OCT measurements, standard errors and p-values of observed associations. The table includes significant linear regression results for 32,113 unrelated participants from UK Biobank participants. Models were adjusted for sex, age, spherical equivalent, smoking status.

Collectively, AMD risk associated genes were strongly associated with many different parameters of outer-retinal layer thickness. ISOS-RPE central thickness was most significantly associated with a cumulative polygenic risk score, composed of AMD-associated variants, resulting in a decreased thickness (beta = -− 0.52 p = 1.37 × 10^–67^). The inclusion of age, sex and smoking status only marginally improved the model’s predictive capacity. However, AMD-associated genes appear to be only a minor predictor of SD-OCT measurement variability, especially compared to other factors such as smoking, age (Supplemental Table S4) and sex. The AMD polygenic risk score only explained 0.2–6% of SD-OCT measurements variability (Table 3). Generally increasing AMD-risk PRS was very significantly associated with a reduction in the thickness of layers that included the retinal pigmented epithelium layers (ISOS-RPE and RPE-BM, both central and average measurements) and, much less statistically significantly, with a thickening of layers that were defined in a way that included the external limiting membrane (INL-ELM and ELM-ISOS, both central and average measurements).

**Table 3:** SD-OCT variance predicted by a model adjusted by for sex, age, spherical equivalent, smoking status and polygenic risk score (PRS).

Measurement	Beta	SE	p-value	PRS model R2	Null model R2
ELM-ISOS (average)	0.02	0.01	0.01	0.0668	0.0666
ELM-ISOS (center)	0.05	0.01	2.39 × 10^–06^	0.0189	0.0183
INL-ELM (average)	0.10	0.03	0.004	0.0559	0.0556
INL-ELM (center)	0.14	0.05	0.006	0.0163	0.0160
ISOS-RPE (average)	− 0.18	0.02	3.46 × 10^–20^	0.0038	0.0012
ISOS-RPE (center)	− 0.52	0.03	1.37 × 10^–67^	0.0191	0.0098
RPE-BM (average)	− 0.18	0.02	7.21 × 10^–34^	0.0420	0.0376
RPE-BM (center)	− 0.15	0.02	9.58 × 10^–11^	0.0390	0.0378

The model included SD-OCT measurements as dependent variables and PRS as an independent predictor. PRS was calculated using 33 AMD-associated variants. Columns “Beta”, “SE” and “p-value” show the changes in the measurements per standard deviation in PRS dosage increase, and standard errors and p-values of the associations. Fields “R^2^ (Null model)”, “R^2^ (Model adjusted for PRS)” and “R2 difference” include R^2^ of the null model, adjusted for sex, age, spherical equivalent and smoking status, and the model additionally adjusted for PRS, and the R^2^ difference between null model and the model adjusted for PRS.

**Figure 3:**
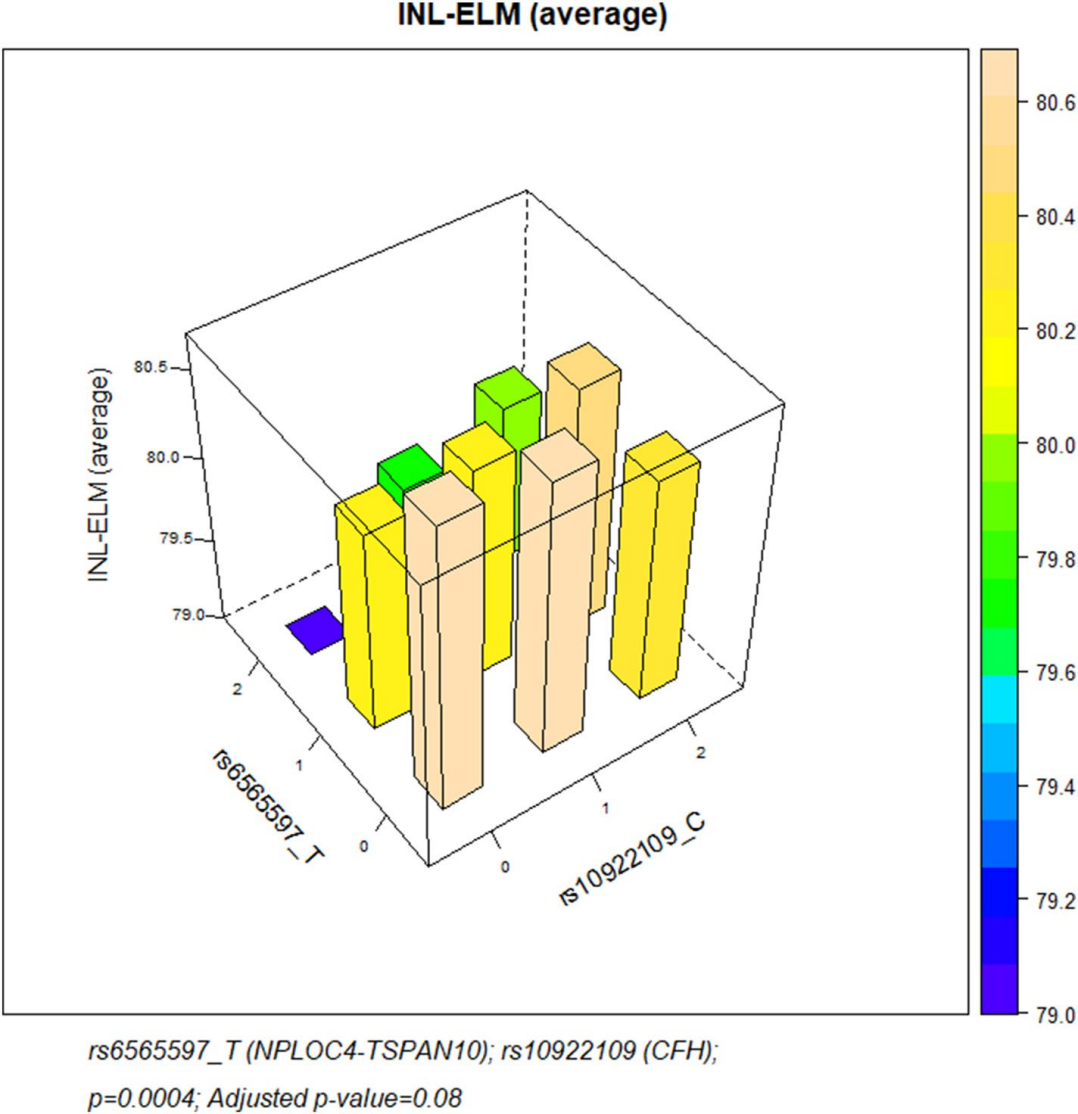
Association of the INL-ELM thickness with two common AMD-associated genetic polymorphisms (rs10922109 and rs6565597). The dosage of the AMD-predisposing alleles at these SNP loci (C and T respectively) is shown in the x- and y-axes. Values of 0, 1 and 2 denote number of the AMD risk alleles present in individual genotypes (i.e. risk allele is not present, heterozygous or homozygous genotypes for that allele respectively). The average INL-ELM thickness for each combination of these genotypes is given in the z-axis (for the sake of clarity the minimum was set at 79.0 microns).

We carried forward the SNPs that were previously associated with any parameter underlying the average and central outer-retinal layer thickness measurements respectively, and specifically looked for non-linear gene by gene interactions of pairs of SNPs. Results for all gene–gene interactions on outer retinal layer thickness can be seen in Table 4. Interactions involving the *NPLOC4-TSPAN10* SNP rs6565597 appeared to be associated with significant outer retinal layer thickness changes. Notably, the interaction between AMD-predisposing alleles *NPLOC4-TSPAN10* (rs6565597.T) and *CFH* (rs10922109.C) significantly increased the average thickness of the INL-ELM (Fig. 3) (beta = 0.25; p = 0.0004). However, this association wasn’t significant after correcting for multiple testing.

**Table 4:** Results of linear models testing the effects of 10 most significant AMD SNP interactions on SD-OCT measurements.

Variants	Genes	Beta	SE	p-value	Measurement	Adjusted p-value
rs10922109_C × rs6565597_T	CFH NPLOC4-TSPAN10	0.25	0.072	0.0004	INL-ELM (average)	0.08
rs6565597_T × rs61985136_T	NPLOC4-TSPAN10 RAD51B	− 0.23	0.068	0.0009	ISOS-RPE (center)	0.16
rs429608_G × rs61985136_T	PBX2 RAD51B	− 0.15	0.058	0.008	ISOS-RPE (average)	1
rs3750846_C × rs429608_G	ARMS2 PBX2	0.29	0.113	0.01	INL-ELM (average)	1
rs6565597_T × rs61985136_T	NPLOC4-TSPAN10 RAD51B	− 0.11	0.045	0.01	ISOS-RPE (average)	1
rs10922109_C × rs6565597_T	CFH NPLOC4-TSPAN10	0.28	0.114	0.01	INL-ELM (center)	1
rs61985136_T × rs429608_G	RAD51B PBX2	− 0.21	0.087	0.02	ISOS-RPE (center)	1
rs6565597_T × rs10033900_T	NPLOC4-TSPAN10 CFI	− 0.12	0.051	0.02	RPE-BM (center)	1
rs3750846_C × rs943080_T	ARMS2 VEGFA	− 0.05	0.019	0.01	ELM-ISOS (average)	1
rs429608_G × rs943080_T	PBX2 VEGFA	0.22	0.092	0.01	INL-ELM (average)	1

Models were adjusted for sex, age, spherical equivalent, smoking status. Field “Interactions” displays rs ID numbers and the risk alleles of the variants included in the interactions, that were nominally associated with outer-retinal layers. The column “Measurements” lists SD-OCT measurements that were tested. The columns “Beta”, “SE”, “p-value” and “Adjusted p-value” denote the change of SD-OCT measurements (in microns), standard errors, 95% confidence intervals, p-values and Bonferroni-corrected p-values for each tested interaction. The table includes results for 32,113 participants from UK Biobank participants.

Conversely, we also found that the interaction between AMD-predisposing rs6565597 T allele (*NPLOC4-TSPAN10*) and the rs61985136 T allele (*RAD51B*) was nominally associated with a decreased central thickness of the ISOS-RPE layer, however, this was not significant after Bonferroni correction (beta = − 0.23, p = 0.0009, *Bonferroni-adjusted p* = 0.15*).*

### Mendelian randomization

To explore the causality of the observed associations, we performed Mendelian randomization (MR) analyses. Our study found evidence for directional pleiotropic relationships between AMD risk and measurements of the outer retinal layer thickness parameters. These results reveal that predisposition to AMD caused a significant decrease in the thickness of the central area of the ISOS-RPE layer (MR-Egger p = 0.04) with no evidence of pleiotropy (MR-Egger intercept p = 0.36). There was less significant evidence for association with the measurements of other outer retinal layers in our dataset (Supplementary Table S5).

## Discussion

This is the first study to analyse the relationship between AMD genetic polymorphisms and changes in outer retinal layer thickness in a large normal population. We focused our analysis on the outer retina as our hypothesis is that AMD related genes would affect photoreceptor and RPE thickness. Our results show a clear but complex relationship between outer retinal thickness and the presence of AMD risk SNPs. By analysing changes in individual retinal layers instead of overall retinal thickness, a greater understanding of possible structural changes can be derived. Importantly, by examining normal individuals and not those with AMD we can identify structural changes that may precede disease development. Mendelian randomization modelling with AMD risk polymorphisms revealed a statistically significant decrease in the ISOS-RPE thickness. This same pattern was also seen when retinal thickness was modelled using cumulative AMD risk in the form of a PRS. Importantly this pattern was seen after adjusting for age, sex, spherical equivalent and smoking status, all of which can influence retinal thickness^26,30^. Consequently, it can be inferred that AMD risk is causally associated with thinner ISOS-RPE measurements in the AMD diagnosis-free general population. One possible explanation for this is that AMD pathological processes begin much earlier in life with ISOS-RPE thinning as an early sign of disease.

The ISOS-RPE measurement is a proxy measure for the photoreceptor outer-segment (POS). Although there is still some debate on the anatomical correlates of the hyperreflective bands observed on OCT imaging^31^. We saw significant thinning of this measurement centrally with the presence of AMD risk SNPs. The POS contains several hundred infolded plasma membrane discs that house visual pigments. The length of the POS reduces as a result of inflammation and oxidative stress^32^, therefore POS length can be used as a marker of photoreceptor health. ISOS-RPE thinning was the most highly significant change seen in our PRS analysis with presence of AMD risk SNPs. POS shortening has been documented in normal fellow eyes of late AMD patients^33^, and OCT scans in AMD patients show a reduction in photoreceptor layer thickness over time^34^. In the healthy retina, photoreceptors maintain a roughly constant length by continuously generating new outer segments from their base, whilst simultaneously releasing mature outer segments that are then engulfed by the RPE during phagocytosis. A change in POS length could arise from photoreceptor degeneration that occurs prior to photoreceptor death, or alterations of phagocytosis by the RPE. In AMD, rod photoreceptors degenerate prior to cones, especially at the perifovea. Furthermore, early in disease, prior to identifiable AMD fundus features, delayed rod-mediated dark adaptation occurs^35^. A shortening of POS, could result in a disruption to the retinoid cycle and consequently lead to delayed rod-mediated dark adaptation, causing previously unidentified changes in otherwise healthy fundi with POS shortening representing a pre-disease AMD biomarker.

Our PRS analysis also revealed a significant reduction in RPE-BM thickness with the presence of risk AMD SNPs. This is particularly interesting in the context of AMD, given that most clinical and histological signs are first seen in the RPE and BM. BM is breached in neovascular (nAMD), and the deposition of drusenoid deposits between the RPE and BM is one of the earliest signs of AMD. BM also plays a crucial role in photoreceptor maintenance. Interestingly, ageing has been associated with increased thickness of BM^36^. Previous reports state that the RPE-BM measurement becomes thinner with age, thereby suggesting that if the BM thickens then the RPE must significantly thin as we get older^24^. In this study, we cannot resolve whether normal individuals with AMD risk polymorphisms have a prematurely thin RPE layer, or a thinner BM. Our analysis has interestingly shown that, in the presence of high genetic risk for AMD, there is a significant decrease in the thickness of both the ISOS-RPE and the RPE-BM which may suggest that premature RPE thinning could be a major contributory factor.

We found evidence for both additive and non-additive effects of several known AMD genetic loci on outer retinal thickness. Surprisingly, no interactive nor individual effect was found for the common and widely researched AMD risk SNPs rs1061170 (Y402H) at *CFH* nor rs10490924 (A69S) within *ARMS2* on retinal layer thickness. Several other gene–gene interactions were identified, all of which involved the *NPLOC4-TSPAN10* SNP rs6565597. We found a statistically significant increase in the INL-ELM average thickness in the presence of AMD-predisposing alleles at the *CFH* (rs10922109) and *NPLOC4-TSPAN10* (rs6565597) loci. In this additive model homozygosity for the *CFH* protective allele (AA) with the *NPLOC4-TSPAN10* risk allele (TT) resulted in a significant thinning to the INL-ELM. This may evidence the significant influence the *CFH* protective allele has on INL-ELM thickness. The INL-ELM is a proxy measure of the synaptic terminals, axons and the nucleus of the photoreceptors that includes the outer plexiform and outer nuclear layers. In this case AMD-predisposing allele interactions surprisingly resulted in a thicker measurement, opposing the trend towards a decrease seen in the majority of our results.

The rs6565597 SNP is located at the *NPLOC4-TSPAN10* locus; however, expression quantitative trait locus (eQTL) data suggests that the impact of this variant is at *TSPAN10* rather than *NPLOC4*^37^. Data suggests that rs6565597 risk allele homozygous carriers (TT) have a significantly decreased production of TSPAN10 mRNA compared to other alleles of this locus^37^. TSPAN10-encoded proteins are ubiquitous in the eye, and expressed at particularly high levels in the RPE, iris, ciliary body and retinal ganglion cells^38^, but also the RPE and melanocytes within the choroid^38,39^. TSPAN10 belongs to the TspanC8 group of tetraspanins and primarily controls the enzymatic maturation of ADAM10^40^, therefore playing an important role in transmembrane signalling pathways that regulate cell fate and development, such as the Notch pathway^40^. Polymorphisms at *TSPAN10* are associated with significantly reduced thickness at the foveola and reduced thickness in the retinal nerve fibre layer and ganglion cell layer at the macula^41^. In addition to AMD^42^, polymorphisms at this locus are associated with disorders affecting every anatomical structure of the eye, such as myopia^43^, astigmatism^44^ and strabismus^45^, but also hair^46^ and iris^47^ pigmentation.

TSPAN10 is thought to play a role in pigmentation. It is currently unknown whether and how variations in pigmentation pathways, such as those of the *TSPAN10* gene, can influence retinal development and retinal layer thickness. Presence of the AMD risk-associated allele, rs6565597, appeared to generally cause a significant thinning of outer retinal layers in normal individuals in this study. Whether these changes to outer retinal thickness put individuals at an increased risk of AMD is currently not known. AMD, especially in the advanced stages is typically seen in the Caucasian population^48,49^. Macular pigmentation is reported to differ between ethnicities^50^, and AMD is known to cause both a reduction in pigment density and retinal thinning^33,51^. If individuals with the TSPAN10 AMD risk variant have a change in macular pigmentation, this could predispose to AMD, eventually causing dysfunction and a premature thinning of the RPE.

AMD is typically a disease of the elderly, with RPE cell loss and atrophy seen early in the disease process. Oxidative stress-induced injury to the RPE is known to result in a chronic inflammatory response, drusen formation, and eventual RPE atrophy. This RPE degeneration in turn causes a progressive degeneration of photoreceptors, leading to the irreversible loss of vision. Our analysis revealed thinning of both the layer representing the POS and that representing the RPE in the presence of AMD risk SNPs. Subsequently, it is highly likely that individuals with seemingly normal ocular examinations, but who are carriers of AMD risk alleles may have previously unidentified changes to these layers prior to symptom onset.

One limitation of our study is the potential misclassification of AMD status. The average age of the Biobank cohort is 56.58 ± 7.98 years; therefore, there may be some individuals with early fundal signs of AMD who were not aware at the time of recruitment. Further, it would have been interesting to know if any of our participations had a family history of AMD but this information was not available in the UK biobank. Therefore we consider the population studied “normal” in the population-based sense, where AMD or other retinopathies are expected to be exceedingly rare and unlikely to change the properties of population distribution which were the main object of our study.

As detailed in Fig. 2, after applying our exclusion criteria 32,113 people were available from an initial 67,139 participants. 15,165 were excluded due to either low signal strength or low quality of the scans. However, due to the remaining large number of participants we believe our results remain robust.

In conclusion, our study demonstrates an association between outer retinal layer thickness and the presence of risk AMD SNPs in the normal population. We report novel findings demonstrating a thinner ISOS-RPE with the presence of AMD risk SNPs and complex additive effects of risk SNPs significantly regulating the retinal layer thickness. Our study highlights the premorbid influence of AMD genetic risk variants on macular thickness and may provide mechanistic insight into the pathophysiology of this debilitating disease.

## Supplementary Information

Supplementary Information.
