# Supplementary material for: Macular thickness varies with age-related macular degeneration genetic risk variants in the UK Biobank cohort
Source: Sci Rep. 2021 Dec 1;11:23255. doi: 10.1038/s41598-021-02631-2 (PMC8636487; doi:10.1038/s41598-021-02631-2)
Supplement: Supplementary file 1 — Supplementary Information. [file 41598_2021_2631_MOESM1_ESM.docx]

**Supplementary Tables & Figures**

**Supplementary Table S1. Characteristics of the study participants** **(N=32,113).**

The fields "(%,N)" and (mean (SD)) denote percentage, number of study participants, means and standard deviations respectively. SE, standard error.

| **Characteristics** | **Statistics** |
| --- | --- |
| *Demographic characteristics* |  |
| Sex (%, N) |  |
| Women | 51.97 % (16,688) |
| Men | 48.03 % (15,425) |
| Age (mean (SD)) | 56.6 (8) |
| SE | 0.02 (1.95) |
| Smoking status (%, N) |  |
| Never-smokers | 54.12 % (17,378) |
| Ever-smokers | 45.88 % (14,735) |
| *SD-OCT measurements* |  |
| INL-ELM (center) | 108.9 (9.4) |
| INL-ELM (average) | 80.9 (6.1) |
| ELM-ISOS (center) | 28.4 (1.8) |
| ELM-ISOS (average) | 23.6 (1.4) |
| ISOS-RPE (center) | 42.9 (5.4) |
| ISOS-RPE (average) | 38.2 (3.5) |
| RPE_BM (center) | 26.1 (4.3) |
| RPE_BM (average) | 25.3 (2.8) |

**Supplementary Figure S1**. **Distributions of SD-OCT measurements in the UK Biobank cohort.**

**A-H** Depict bar charts showing the distribution of outer retinal thickness measurements in the UK Biobank cohort. The distribution of outer retinal thickness measurements for INL-ELM, ELM-ISOS and ISOS-RPE, resembled a normal distribution; however, RPE-BM thickness (A & E) followed a leptokurtic distribution.


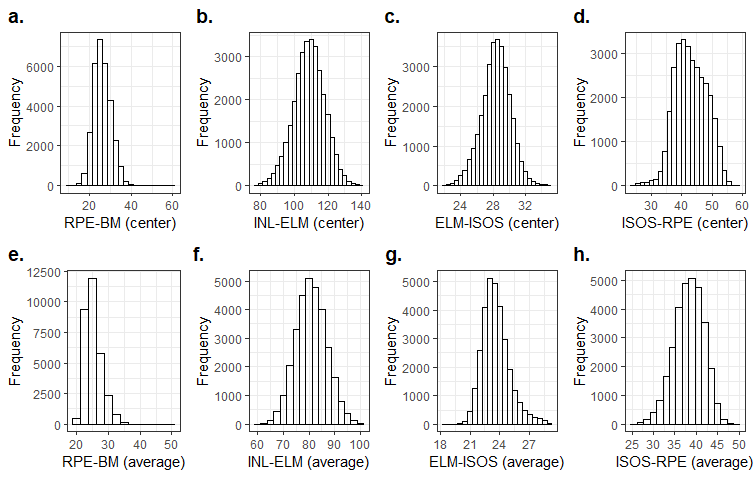


**Supplementary Table S2. Associations between average spectral-domain optical coherence tomography (SD-OCT) measurements and risk AMD variants.** Columns “Variant”, “EA” and “NEA” list variants that were included in the model, and their risk alleles (EA, effect alleles for which the effect sizes are reported and NEA, non-effect alleles). Fields “Beta”, “SE” and “P” denote the change in SD-OCT measurements, standard errors and p-values of observed associations. The table includes significant linear regression results for 32,113 unrelated participants from UK Biobank participants. Models were adjusted for sex, age, spherical equivalent, smoking status

|  |  |  |  |  |  | *ELM-ISOS* | | | *INL-ELM* | | | *ISOS-RPE* | | | | *RPE-BM* | | | |
| --- | --- | --- | --- | --- | --- | --- | --- | --- | --- | --- | --- | --- | --- | --- | --- | --- | --- | --- | --- |
| **Chr** | **BP** | **Variant** | **Gene** | **EA** | **NEA** | **Beta** | **SE** | **p-value** | **Beta** | **SE** | **p-value** | **Beta** | **SE** | **p-value** | **Beta** | | **SE** | **p-value** |  |
| 1 | 196704632 | rs10922109 | CFH | C | A | 0.02 | 0.01 | 0.1 | -0.036 | 0.048 | 0.5 | **-0.26** | **0.03** | **6.63x10^-20^** | **-0.322** | | **0.022** | **1.47x10^-49^** |  |
| 2 | 228086920 | rs11884770 | COL4A3 | C | T | -0.01 | 0.01 | 0.3 | 0.065 | 0.053 | 0.2 | -0.01 | 0.03 | 0.7 | 0.028 | | 0.024 | 0.2 |  |
| 3 | 99180668 | rs140647181 | COL8A1 | C | T | -0.02 | 0.04 | 0.7 | 0.458 | 0.193 | 0.02 | -0.27 | 0.12 | 0.02 | 0.094 | | 0.088 | 0.3 |  |
| 3 | 64715155 | rs62247658 | ADAMTS9 | C | T | 0.01 | 0.01 | 0.3 | 0.034 | 0.048 | 0.5 | 0.009 | 0.03 | 0.8 | -0.046 | | 0.022 | 0.03 |  |
| 4 | 110659067 | rs10033900 | CFI | T | C | 0.001 | 0.01 | 0.9 | -0.002 | 0.047 | 1 | -0.08 | 0.03 | 0.003 | -0.012 | | 0.021 | 0.6 |  |
| 5 | 35494448 | rs114092250 | SPEF2 | G | A | 0.01 | 0.03 | 0.7 | 0.135 | 0.146 | 0.4 | -0.09 | 0.09 | 0.3 | 0.021 | | 0.067 | 0.8 |  |
| 5 | 39327888 | rs62358361 | C9 | T | G | 0.1 | 0.05 | 0.1 | 0.3 | 0.227 | 0.2 | -0.1 | 0.14 | 0.5 | -0.101 | | 0.104 | 0.3 |  |
| 6 | 31930462 | rs429608 | PBX2 | G | A | 0.02 | 0.01 | 0.1 | 0.017 | 0.065 | 0.8 | **-0.19** | **0.04** | **6.08x10^-07^** | -0.08 | | 0.03 | 0.01 |  |
| 6 | 43826627 | rs943080 | VEGFA | T | C | -0.00 | 0.01 | 0.8 | -0.048 | 0.047 | 0.3 | 0.005 | 0.03 | 0.9 | **-0.084** | | **0.021** | **8.83x10^-05^** |  |
| 7 | 104756326 | rs1142 | KMT2E | T | C | -0.00 | 0.01 | 0.8 | -0.026 | 0.049 | 0.6 | -0.03 | 0.03 | 0.3 | -0.024 | | 0.022 | 0.3 |  |
| 7 | 99991548 | rs7803454 | PILRA | T | C | 0.03 | 0.01 | 0 | 0.199 | 0.059 | 0.0008 | **0.245** | **0.04** | **5.15x10^-12^** | **-0.107** | | **0.027** | **7.67x10^-05^** |  |
| 8 | 23082971 | rs13278062 | TNFRSF10A | T | G | -0.01 | 0.01 | 0.4 | 0.023 | 0.047 | 0.62 | 0.005 | 0.03 | 0.8 | 0.006 | | 0.022 | 0.8 |  |
| 9 | 76617720 | rs10781182 | RORB | T | G | 0.00 | 0.01 | 1 | **0.215** | **0.051** | **2.11x10^-05^** | -0.05 | 0.03 | 0.1 | 0.024 | | 0.023 | 0.3 |  |
| 9 | 101923372 | rs1626340 | TGFBR1 | G | A | 0.02 | 0.01 | 0.2 | 0.055 | 0.057 | 0.3 | 0.065 | 0.03 | 0.06 | 0.001 | | 0.026 | 1 |  |
| 9 | 107661742 | rs2740488 | ABCA1 | A | C | -0.01 | 0.01 | 0.7 | -0.059 | 0.053 | 0.3 | -0.04 | 0.03 | 0.2 | 0.014 | | 0.024 | 0.6 |  |
| 9 | 73438605 | rs71507014 | TRPM3 | G | GC | 0.01 | 0.01 | 0.2 | 0.121 | 0.048 | 0.01 | 0.035 | 0.03 | 0.23 | 0.027 | | 0.022 | 0.2 |  |
| 10 | 24999593 | rs12357257 | ARHGAP21 | A | G | 0.01 | 0.01 | 0.6 | 0.045 | 0.057 | 0.4 | -0.04 | 0.03 | 0.21 | 0.012 | | 0.026 | 0.6 |  |
| 10 | 124215565 | rs3750846 | ARMS2 | C | T | 0.01 | 0.01 | 0.3 | 0.074 | 0.057 | 0.2 | **-0.26** | **0.03** | **5.32x10^-14^** | -0.068 | | 0.026 | 0.01 |  |
| 12 | 56115778 | rs3138141 | BLOC1S1-RDH5 | A | C | **0.05** | **0.01** | **0.0001** | **0.824** | **0.053** | **3.34x10^-54^** | **0.389** | **0.03** | **2.44x10^-34^** | **0.091** | | **0.024** | **0.0002** |  |
| 12 | 112132610 | rs61941274 | ACAD10 | A | G | -0.07 | 0.03 | 0.03 | 0.222 | 0.15 | 0.1 | 0.289 | 0.09 | 1.27E-03 | -0.169 | | 0.069 | 0.01 |  |
| 13 | 31821240 | rs9564692 | B3GALTL | C | T | -0.01 | 0.01 | 0.5 | 0.055 | 0.052 | 0.3 | 0.005 | 0.03 | 0.9 | 0.014 | | 0.024 | 0.6 |  |
| 14 | 68769199 | rs61985136 | RAD51B | T | C | 0.03 | 0.01 | 0.02 | **0.223** | **0.049** | **5.35x10^-06^** | 0.038 | 0.03 | 0.2 | -0.052 | | 0.022 | 0.02 |  |
| 15 | 58680954 | rs2043085 | LIPC | C | T | -0 | 0.01 | 0.8241 | -0.061 | 0.048 | 0.2 | 0.014 | 0.03 | 0.6 | -0.069 | | 0.022 | 0.002 |  |
| 16 | 56997349 | rs5817082 | CETP | C | CA | 0.02 | 0.01 | 0.2 | -0.048 | 0.053 | 0.4 | -0.08 | 0.03 | 1.09E-02 | 0.017 | | 0.024 | 0.5 |  |
| 16 | 75234872 | rs72802342 | CTRB2 | C | A | -0.01 | 0.02 | 0.6 | -0.141 | 0.09 | 0.1 | -0.08 | 0.05 | 0.13 | 0.021 | | 0.041 | 0.6 |  |
| 17 | 26649724 | rs11080055 | TMEM97 | C | A | -0 | 0.01 | 0.9 | 0.025 | 0.047 | 0.6 | 0.01 | 0.03 | 0.73 | 0.01 | | 0.021 | 0.7 |  |
| 17 | 79526821 | rs6565597 | NPLOC4-TSPAN10 | T | C | -0.04 | 0.01 | 0.0008 | **-0.374** | **0.05** | **8.76x10^-14^** | **0.377** | **0.03** | **4.25x10^-36^** | **-0.3** | | **0.023** | **1.00x10^-38^** |  |
| 19 | 6718387 | rs2230199 | C3 | G | C | -0.02 | 0.01 | 0.2 | -0.061 | 0.058 | 0.3 | **0.16** | **0.04** | **5.04x10^-06^** | 0.042 | | 0.027 | 0.1 |  |
| 19 | 45411941 | rs429358 | APOE | T | C | -0.02 | 0.01 | 0.3 | 0.104 | 0.065 | 0.1 | -0.05 | 0.04 | 0.2 | 0.022 | | 0.03 | 0.5 |  |
| 19 | 1031438 | rs67538026 | CNN2 | C | T | 0 | 0.01 | 0.8 | 0.032 | 0.049 | 0.5 | -0.05 | 0.03 | 0.1 | 0.015 | | 0.022 | 0.5 |  |
| 20 | 56653724 | rs201459901 | C20orf85 | T | TA | 0 | 0.02 | 0.9 | 0.26 | 0.102 | 0.01 | -0.08 | 0.06 | 0.2 | 0.075 | | 0.047 | 0.1 |  |
| 22 | 33105817 | rs5754227 | SYN3 | T | C | 0.03 | 0.02 | 0.1 | -0.012 | 0.07 | 0.9 | -0.09 | 0.04 | 0.04 | 0.084 | | 0.032 | 0.01 |  |
| 22 | 38476276 | rs8135665 | SLC16A8 | T | C | 0.01 | 0.01 | 0.3 | 0.084 | 0.059 | 0.2 | -0 | 0.04 | 0.94 | -0.057 | | 0.027 | 0.04 |  |

**Supplementary Table S3.** **Associations between central spectral-domain optical coherence tomography (SD-OCT) measurements and risk AMD variants.** Columns “Variant”, “EA” and “NEA” list variants that were included in the model, and their risk alleles (EA, effect alleles for which the effect sizes are reported and NEA, non-effect alleles). Fields “Beta”, “SE” and “P” denote the change in SD-OCT measurements, standard errors and p-values of observed associations. The table includes significant linear regression results for 32,113 unrelated participants from UK Biobank participants. Models were adjusted for sex, age, spherical equivalent, smoking status.

|  |  |  |  |  |  | *ELM-ISOS* | | | *INL-ELM* | | | *ISOS-RPE* | | | *RPE-BM* | | |
| --- | --- | --- | --- | --- | --- | --- | --- | --- | --- | --- | --- | --- | --- | --- | --- | --- | --- |
| **Chr** | **BP** | **Variant** | **Gene** | **EA** | **NEA** | **Beta** | **SE** | **P** | **Beta** | **SE** | **P** | **Beta** | **SE** | **P** | **Beta** | **SE** | **P** |
| 1 | 196704632 | rs10922109 | CFH | C | A | **0.061** | **0.015** | **2.94x10^-05^** | 0.016 | 0.075 | 0.8 | **-0.601** | **0.043** | **3.45x10^-44^** | **-0.330** | **0.034** | **5.02x10^-22^** |
| 2 | 228086920 | rs11884770 | COL4A3 | C | T | -0.013 | 0.016 | 0.4 | 0.089 | 0.084 | 0.3 | -0.018 | 0.048 | 0.7 | 0.043 | 0.038 | 0.3 |
| 3 | 99180668 | rs140647181 | COL8A1 | C | T | 0.052 | 0.059 | 0.4 | 0.497 | 0.305 | 0.1 | -0.474 | 0.175 | 0.01 | 0.186 | 0.139 | 0.2 |
| 3 | 64715155 | rs62247658 | ADAMTS9 | C | T | 0.016 | 0.015 | 0.3 | -0.022 | 0.075 | 0.8 | 0.073 | 0.043 | 0.09 | -0.048 | 0.034 | 0.2 |
| 4 | 110659067 | rs10033900 | CFI | T | C | 0.025 | 0.014 | 0.08 | 0.023 | 0.074 | 0.8 | **-0.168** | **0.042** | **7.50x10^-05^** | 0.032 | 0.034 | 0.3 |
| 5 | 35494448 | rs114092250 | SPEF2 | G | A | -0.026 | 0.045 | 0.6 | 0.239 | 0.231 | 0.3 | -0.332 | 0.133 | 0.01 | 0.207 | 0.105 | 0.05 |
| 5 | 39327888 | rs62358361 | C9 | T | G | 0.108 | 0.070 | 0.1 | 0.168 | 0.358 | 0.6 | -0.297 | 0.206 | 0.1 | 0.085 | 0.163 | 0.6 |
| 6 | 31930462 | rs429608 | PBX2 | G | A | 0.012 | 0.020 | 0.6 | -0.101 | 0.102 | 0.3 | **-0.450** | **0.059** | **1.92x10^-14^** | -0.030 | 0.047 | 0.5 |
| 6 | 43826627 | rs943080 | VEGFA | T | C | 0.027 | 0.014 | 0.06 | 0.028 | 0.074 | 0.7 | -0.102 | 0.042 | 0.02 | 0.047 | 0.034 | 0.2 |
| 7 | 99991548 | rs7803454 | PILRA | T | C | -0.017 | 0.018 | 0.4 | 0.187 | 0.094 | 0.05 | 0.127 | 0.054 | 0.02 | **-0.190** | **0.043** | **8.38x10^-06^** |
| 7 | 104756326 | rs1142 | KMT2E | T | C | -0.014 | 0.015 | 0.4 | -0.087 | 0.077 | 0.3 | -0.156 | 0.044 | 0.0004 | 0.010 | 0.035 | 0.8 |
| 8 | 23082971 | rs13278062 | TNFRSF10A | T | G | 0.019 | 0.014 | 0.2 | 0.072 | 0.074 | 0.3 | -0.012 | 0.043 | 0.8 | 0.019 | 0.034 | 0.6 |
| 9 | 73438605 | rs71507014 | TRPM3 | G | GC | -0.016 | 0.015 | 0.3 | 0.027 | 0.076 | 0.7 | 0.054 | 0.044 | 0.2 | 0.052 | 0.035 | 0.1 |
| 9 | 76617720 | rs10781182 | RORB | T | G | 0.012 | 0.016 | 0.4 | 0.107 | 0.080 | 0.2 | -0.100 | 0.046 | 0.03 | 0.046 | 0.036 | 0.2 |
| 9 | 101923372 | rs1626340 | TGFBR1 | G | A | -0.016 | 0.018 | 0.4 | 0.020 | 0.090 | 0.8 | 0.114 | 0.052 | 0.03 | 0.002 | 0.041 | 1.0 |
| 9 | 107661742 | rs2740488 | ABCA1 | A | C | 0.015 | 0.016 | 0.4 | 0.053 | 0.084 | 0.5 | -0.054 | 0.048 | 0.3 | 0.043 | 0.038 | 0.3 |
| 10 | 24999593 | rs12357257 | ARHGAP21 | A | G | 0.015 | 0.017 | 0.4 | 0.028 | 0.090 | 0.8 | -0.118 | 0.051 | 0.02 | 0.039 | 0.041 | 0.3 |
| 10 | 124215565 | rs3750846 | ARMS2 | C | T | 0.047 | 0.018 | 0.01 | 0.102 | 0.090 | 0.3 | **-0.682** | **0.052** | **2.36x10^-39^** | 0.011 | 0.041 | 0.8 |
| 12 | 56115778 | rs3138141 | BLOC1S1-RDH5 | A | C | -0.032 | 0.016 | 0.05 | **1.178** | **0.084** | **1.22x10^-44^** | **0.686** | **0.048** | **7.21x10^-46^** | 0.129 | 0.038 | 0.001 |
| 12 | 112132610 | rs61941274 | ACAD10 | A | G | -0.018 | 0.046 | 0.7 | 0.630 | 0.237 | 0.01 | 0.402 | 0.136 | 0.003 | -0.078 | 0.108 | 0.5 |
| 13 | 31821240 | rs9564692 | B3GALTL | C |  | 0.004 | 0.016 | 0.8 | 0.031 | 0.082 | 0.7 | -0.036 | 0.047 | 0.4 | 0.033 | 0.037 | 0.4 |
| 14 | 68769199 | rs61985136 | RAD51B | T | C | 0.048 | 0.015 | 0.001 | **0.324** | **0.077** | **2.93x10^-05^** | 0.023 | 0.045 | 0.6 | -0.027 | 0.035 | 0.4 |
| 15 | 58680954 | rs2043085 | LIPC | C | T | 0.006 | 0.015 | 0.7 | -0.127 | 0.076 | 0.09 | -0.001 | 0.044 | 1.0 | -0.031 | 0.035 | 0.4 |
| 16 | 56997349 | rs5817082 | CETP | C | CA | 0.019 | 0.016 | 0.2 | -0.006 | 0.084 | 0.9 | -0.140 | 0.048 | 0.004 | -0.007 | 0.038 | 0.9 |
| 16 | 75234872 | rs72802342 | CTRB2 | C | A | -0.005 | 0.028 | 0.9 | -0.117 | 0.142 | 0.4 | -0.173 | 0.082 | 0.03 | 0.009 | 0.065 | 0.9 |
| 17 | 26649724 | rs11080055 | TMEM97 | C | A | 0.014 | 0.014 | 0.3 | -0.121 | 0.074 | 0.1 | 0.003 | 0.042 | 0.9 | -0.048 | 0.034 | 0.2 |
| 17 | 79526821 | rs6565597 | NPLOC4-TSPAN10 | T | C | **-0.061** | **0.015** | **8.08x10^-05^** | **-0.388** | **0.079** | **9.67x10^-07^** | **0.407** | **0.046** | **4.20x10^-19^** | **-0.509** | **0.036** | **5.12x10^-45^** |
| 19 | 6718387 | rs2230199 | C3 | G | C | -0.029 | 0.018 | 0.1 | -0.068 | 0.092 | 0.5 | **0.275** | **0.053** | **2.15x10^-07^** | 0.003 | 0.042 | 0.9 |
| 19 | 45411941 | rs429358 | APOE | T | C | -0.010 | 0.020 | 0.6 | 0.035 | 0.102 | 0.7 | -0.206 | 0.059 | 0.0004 | -0.025 | 0.047 | 0.6 |
| 19 | 1031438 | rs67538026 | CNN2 | C | T | 0.000 | 0.015 | 1.0 | 0.155 | 0.077 | 0.05 | -0.073 | 0.044 | 0.1 | 0.006 | 0.035 | 0.9 |
| 20 | 56653724 | rs201459901 | C20orf85 | T | TA | 0.062 | 0.031 | 0.05 | 0.326 | 0.161 | 0.04 | -0.025 | 0.092 | 0.8 | 0.094 | 0.073 | 0.2 |
| 22 | 33105817 | rs5754227 | SYN3 | T | C | 0.016 | 0.021 | 0.5 | 0.065 | 0.110 | 0.6 | -0.101 | 0.063 | 0.1 | 0.054 | 0.050 | 0.3 |
| 22 | 38476276 | rs8135665 | SLC16A8 | T | C | 0.021 | 0.018 | 0.2 | 0.175 | 0.093 | 0.06 | 0.005 | 0.053 | 0.9 | -0.118 | 0.042 | 0.01 |

**Supplementary Table S4. Association between SD-OCT measurements and age**. Fields “Beta”, “SE” and “P” denote the change in SD-OCT measurements (µm change per year), standard errors and p-values of observed associations

|  | **Beta** | **SE** | **p-value** |
| --- | --- | --- | --- |
| INL-ELM (average) | 0.05006 | 0.006589 | 3.13 x10^-14^ |
| INL-ELM (center) | 0.003 | 0.0042 | 0.42 |
| ELM-ISOS (center) | -0.021 | 0.0013 | 1.45 x 10^-61^ |
| ELM-ISOS (average) | -0.036 | 0.000972 | 4.49 x 10^-288^ |
| ISOS-RPE (center) | -0.062 | 0.004 | 1.98 x 10^-61^ |
| ISOS-RPE (average) | -0.002 | 0.002 | 0.34 |
| RPE-BM (center) | -0.081 | 0.003 | 8.8 x 10^-161^ |
| RPE-BM (average) | -0.043 | 0.002 | 8.94 x 10^-112^ |

**Supplementary Table S5. Results of Mendelian randomisation analyses testing the causal association between AMD and 4 different SD-OCT measurements.** Columns "Exposure" and "Outcome" include the names of the tested traits. Field "Test" lists the names of the tests that were used to assess causality. The columns "Beta", "SE", "95% CI" and "p-value" denote respectively the MR models coefficients, standard errors and 95% confidence intervals computed by each method. The coefficient units denote the difference in SD-OCT measurements between AMD cases and controls.

| **Exposure** | **Outcome** | **Method** | **Estimate** | **SE** | **95% CI** | | **p-value** |
| --- | --- | --- | --- | --- | --- | --- | --- |
| AMD | ISOS-RPE (center) | Simple median | -0.38 | 0.14 | -0.65 | -0.12 | 0.005 |
|  |  | Weighted median | -0.58 | 0.12 | -0.82 | -0.34 | 1.61x10^-06^ |
|  |  | IVW | -0.34 | 0.13 | -0.60 | -0.09 | 0.008 |
|  |  | MR-Egger | -0.60 | 0.30 | -1.18 | -0.01 | 0.04 |
|  |  | (intercept) | 0.04 | 0.04 | -0.05 | 0.13 | 0.35 |
| AMD | ELM-ISOS (center) | Simple median | 0.04 | 0.03 | -0.01 | 0.09 | 0.09 |
|  |  | Weighted median | 0.05 | 0.01 | 0.02 | 0.08 | 0.0003 |
|  |  | IVW | 0.05 | 0.01 | 0.02 | 0.07 | 0.0003 |
|  |  | MR-Egger | 0.05 | 0.02 | 0.02 | 0.08 | 0.003 |
|  |  | (intercept) | 0.00 | 0.01 | -0.01 | 0.01 | 0.64 |
| AMD | INL-ELM (center) | Simple median | 0.24 | 0.14 | -0.03 | 0.52 | 0.08 |
|  |  | Weighted median | 0.08 | 0.07 | -0.05 | 0.21 | 0.22 |
|  |  | IVW | 0.08 | 0.06 | -0.04 | 0.19 | 0.19 |
|  |  | MR-Egger | 0.03 | 0.08 | -0.13 | 0.18 | 0.75 |
|  |  | (intercept) | 0.03 | 0.03 | -0.03 | 0.08 | 0.33 |
| AMD | RPE-BM (center) | Simple median | 0.12 | 0.07 | -0.01 | 0.25 | 0.08 |
|  |  | Weighted median | 0.01 | 0.04 | -0.06 | 0.08 | 0.77 |
|  |  | IVW | 0.03 | 0.04 | -0.04 | 0.10 | 0.40 |
|  |  | MR-Egger | -0.01 | 0.05 | -0.10 | 0.09 | 0.91 |
|  |  | (intercept) | 0.01 | 0.01 | -0.01 | 0.04 | 0.31 |
| AMD | ISOS-RPE (average) | Simple median | -0.15 | 0.07 | -0.29 | -0.02 | 0.03 |
|  |  | Weighted median | -0.14 | 0.07 | -0.28 | 0.00 | 0.05 |
|  |  | IVW | -0.14 | 0.06 | -0.26 | -0.01 | 0.03 |
|  |  | MR-Egger | -0.21 | 0.14 | -0.49 | 0.07 | 0.14 |
|  |  | (intercept) | 0.01 | 0.02 | -0.03 | 0.06 | 0.57 |
| AMD | ELM-ISOS (average) | Simple median | 0.03 | 0.02 | -0.01 | 0.07 | 0.15 |
|  |  | Weighted median | 0.02 | 0.01 | 0.00 | 0.05 | 0.04 |
|  |  | IVW | 0.03 | 0.01 | 0.01 | 0.04 | 0.01 |
|  |  | MR-Egger | 0.02 | 0.01 | 0.00 | 0.05 | 0.06 |
|  |  | (intercept) | 0.00 | 0.00 | -0.01 | 0.01 | 0.92 |
| AMD | INL-ELM (average) | Simple median | 0.23 | 0.09 | 0.05 | 0.41 | 0.01 |
|  |  | Weighted median | 0.05 | 0.05 | -0.04 | 0.15 | 0.29 |
|  |  | IVW | 0.07 | 0.04 | -0.02 | 0.15 | 0.12 |
|  |  | MR-Egger | 0.02 | 0.06 | -0.09 | 0.13 | 0.73 |
|  |  | (intercept) | 0.02 | 0.02 | -0.01 | 0.06 | 0.22 |
| AMD | RPE-BM (average) | Simple median | 0.07 | 0.05 | -0.02 | 0.17 | 0.13 |
|  |  | Weighted median | -0.06 | 0.02 | -0.11 | -0.01 | 0.02 |
|  |  | IVW | -0.04 | 0.03 | -0.11 | 0.02 | 0.16 |
|  |  | MR-Egger | -0.06 | 0.05 | -0.15 | 0.03 | 0.20 |
|  |  | (intercept) | 0.01 | 0.01 | -0.02 | 0.03 | 0.65 |

**Members of the UK Biobank Eye and Vision Consortium**

Prof Tariq ASLAM - Manchester University
Prof Sarah BARMAN - Kingston University
Prof Jenny BARRETT - University of Leeds
Prof Paul BISHOP - Manchester University
Dr Catey BUNCE - King’s College London
Dr Roxana CARARE - University of Southampton
Prof Usha CHAKRAVARTHY - Queens University, Belfast
Miss Michelle CHAN - Moorfields Eye Hospital, London
Dr Valentina CIPRIANI - UCL Institute of Ophthalmology
Dr Alexander DAY - Moorfields Eye Hospital, London
Miss Parul DESAI - Moorfields Eye Hospital, London
Prof Bal DHILLON - University of Edinburgh
Prof Andrew DICK - University of Bristol
Dr Cathy EGAN - Moorfields Eye Hospital, London
Prof Sarah ENNIS - University of Southampton
Prof Paul FOSTER - UCL Institute of Ophthalmology
Dr Marcus FRUTTIGER - UCL Institute of Ophthalmology
Dr John GALLACHER - University of Oxford
Prof David (Ted) GARWAY-HEATH - UCL Institute of Ophthalmology
Dr Jane GIBSON - University of Southampton
Mr Dan GORE - Moorfields Eye Hospital, London
Prof Jeremy GUGGENHEIM - Cardiff University
Prof Chris HAMMOND - King's College London
Prof Alison HARDCASTLE - UCL Institute of Ophthalmology
Prof Simon HARDING - University of Liverpool
Dr Ruth HOGG - Queen's University, Belfast
Mr Pearse A KEANE - UCL Institute of Ophthalmology
Prof Sir Peng Tee KHAW - UCL Institute of Ophthalmology
Dr Anthony KHAWAJA - Moorfields Eye Hospital, London
Mr Gerassimos LASCARATOS - Moorfields Eye Hospital, London
Prof Phil LUTHERT - UCL Institute of Ophthalmology
Dr Tom MACGILLIVRAY - University of Edinburgh
Dr Sarah MACKIE - University of Leeds
Dr Bernadette MCGUINNESS - Queen’s University Belfast
Dr Gareth MCKAY - Queen's University Belfast
Mr Martin MCKIBBIN - Leeds Teaching Hospitals NHS Trust
Dr Danny MITRY - University of Edinburgh
Prof Tony MOORE - UCL Institute of Ophthalmology
Prof James MORGAN - Cardiff University
Ms Zaynah MUTHY - UCL Institute of Ophthalmology
Mr Eoin O'SULLIVAN - King's College Hospital
Dr Chris OWEN - St George's, University of London
Mr Euan PATERSON - Queens University Belfast
Dr Tunde PETO - Queen's University Belfast
Dr Axel PETZOLD - UCL Institute of Neurology
Prof Jugnoo RAHI - UCL Institute of Child Health
Dr Alicja RUDNICKA - St George's, University of London
Mr Jay SELF - University of Southampton
Prof Sobha SIVAPRASAD - Moorfields Eye Hospital, London
Mr David STEEL - Newcastle University
Mrs Irene STRATTON - Gloucestershire Hospitals NHS Foundation Trust
Mr Nicholas STROUTHIDIS - Moorfields Eye Hospital, London
Prof Cathie SUDLOW - University of Edinburgh
Dr Caroline THAUNG - UCL Institute of Ophthalmology
Miss Dhanes THOMAS - Moorfields Eye Hospital, London
Prof Emanuele TRUCCO - University of Dundee
Mr Adnan TUFAIL - Moorfields Eye Hospital, London
Dr Veronique VITART - University of Edinburgh
Prof Stephen VERNON - University Hospital, Nottingham
Mr Ananth VISWANATHAN - Moorfields Eye Hospital, London
Miss Cathy WILLIAMS - University of Bristol
Dr Katie WILLIAMS - King's College London
Prof Jayne WOODSIDE - Queen's University Belfast
Dr Max YATES - University of East Anglia
Ms Jennifer YIP - University of Cambridge
Dr Yalin ZHENG - University of Liverpool
Dr Sharon Chua - UCL Institute of Ophthalmology
Dr Robyn TAPP - St George's, University of London
Dr Denize ATAN - University of Bristol
Dr Alexander DONEY - University of Dundee
Prof Naomi Allen - University of Oxford
Dr Thomas Littlejohns - University of Oxford
Dr Panagiotis Sergouniotis - The University of Manchester
Prof Graeme Black - The University of Manchester
Dr Nikolas Pontikos - UCL Institute of Ophthalmology
